# Ventral—Dorsal Functional Contribution of the Posterior Cingulate Cortex in Human Spatial Orientation: A Meta-Analysis

**DOI:** 10.3389/fnhum.2018.00190

**Published:** 2018-05-08

**Authors:** Ford Burles, Alberto Umiltá, Liam H. McFarlane, Kendra Potocki, Giuseppe Iaria

**Affiliations:** Neurolab, Department of Psychology, Hotchkiss Brain Institute, and Alberta Children’s Hospital Research Institute, University of Calgary, Calgary, AB, Canada

**Keywords:** hippocampus, navigation, retrosplenial, spatial memory, cognitive map

## Abstract

The retrosplenial cortex has long been implicated in human spatial orientation and navigation. However, neural activity peaks labeled “retrosplenial cortex” in human neuroimaging studies investigating spatial orientation often lie significantly outside of the retrosplenial cortex proper. This has led to a large and anatomically heterogenous region being ascribed numerous roles in spatial orientation and navigation. Here, we performed a meta-analysis of functional Magnetic Resonance Imaging (fMRI) investigations of spatial orientation and navigation and have identified a ventral-dorsal functional specialization within the posterior cingulate for *spatial encoding* vs. *spatial recall*. Generally, ventral portions of the posterior cingulate cortex were more likely to be activated by *spatial encoding*, i.e., passive viewing of scenes or active navigation without a demand to respond, perform a spatial computation, or localize oneself in the environment. Conversely, dorsal portions of the posterior cingulate cortex were more likely to be activated by cognitive demands to recall spatial information or to produce judgments of distance or direction to non-visible locations or landmarks. The greatly varying resting-state functional connectivity profiles of the ventral (centroids at MNI −22, −60, 6 and 20, −56, 6) and dorsal (centroid at MNI 4, −60, 28) posterior cingulate regions identified in the meta-analysis supported the conclusion that these regions, which would commonly be labeled as “retrosplenial cortex,” should be more appropriately referred to as distinct subregions of the posterior cingulate cortex. We suggest that future studies investigating the role of the retrosplenial and posterior cingulate cortex in spatial tasks carefully localize activity in the context of these identifiable subregions.

## Introduction

Over a century ago, Korbinian Brodmann published an exhaustive cytological parcellation of the human cerebral cortex (Brodmann, 2006); as a testament to his work, this parcellation is still commonly used across all neurological disciplines. Of particular interest in Brodmann’s parcellation is the retrosplenial cortex (Brodmann’s areas 26, 29, and 30), a small, enigmatic region in the human brain that Brodmann was only able to identify after delineating this region in lower animals, in which it is relatively larger and more easily identifiable (Brodmann, 2006, 124). In humans, the retrosplenial cortex occupies the small portion of the cingulate cortex that is immediately posterior to the most posterior region of the corpus callosum (i.e., the splenium). While at the time Brodmann was unsure of the significance of the retrosplenial cortex (and neighboring posterior cingulate areas 23 and 31; Brodmann, 2006, 123), more recently, this tiny region has been ascribed important functions involving emotion processing (Maddock, 1999) and episodic memory (Spreng et al., 2009), with substantial literature reporting its critical role in spatial orientation and navigation (Aguirre and D’Esposito, 1999; Maguire, 2001; Vann et al., 2009; Epstein et al., 2017).

**Figure 1:**
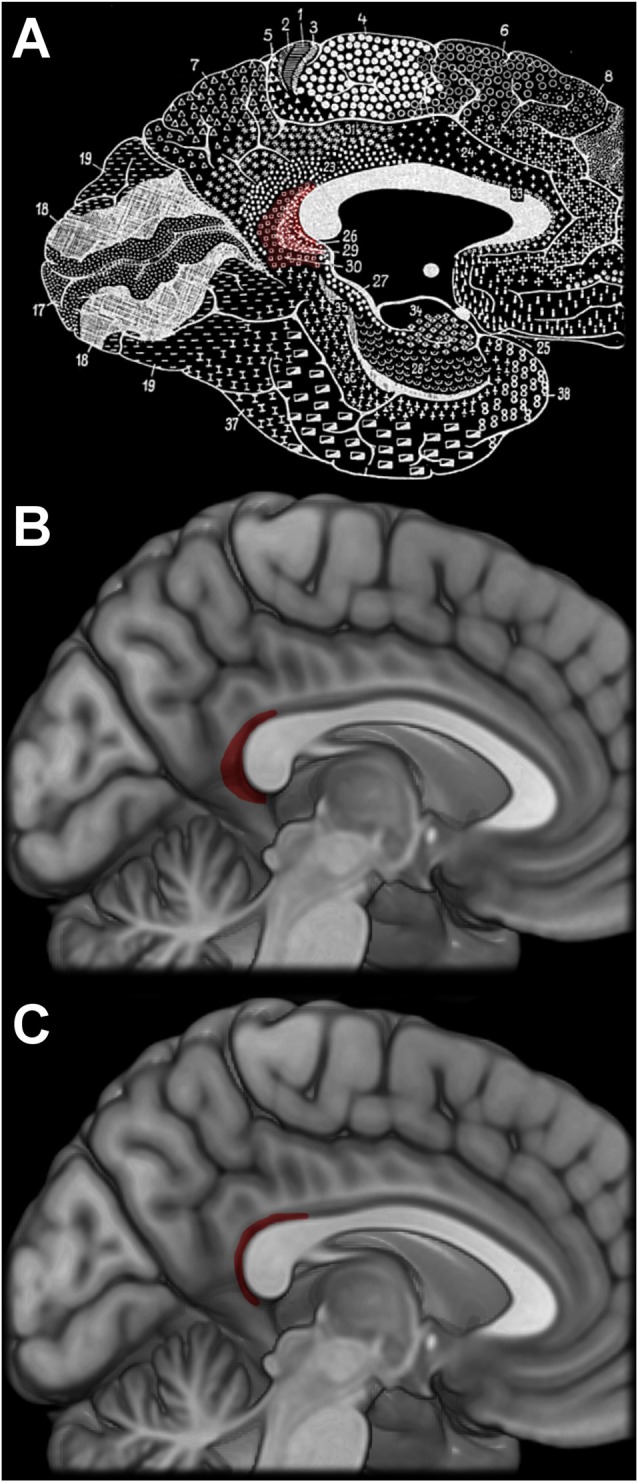
Brodmann’s original depiction of the retrosplenial cortex **(A)**, which was intentionally overrepresented (Brodmann, 2006). More modern illustrations based off the work by Morris et al. (2000) and Vogt et al. (2001) in panels **(B,C)**, respectively, depict a substantially humbler region. Brodmann’s figures, originally published in 1910, are in the public domain.

Despite a precise localization of the retrosplenial cortex in the human brain, the vast majority of functional Magnetic Resonance Imaging (fMRI) studies investigating the role of this region in spatial cognition do not report results in the retrosplenial cortex proper. This is partially due to the fact that the retrosplenial cortex, as delineated by Vogt et al. (2001; Figure 1C) is effectively too small to be studied with common fMRI voxel sizes (e.g., 3 mm isotropic) used for whole-brain imaging, resulting in many fMRI peaks labeled as “retrosplenial cortex” lying in the posterior cingulate cortex (Vogt et al., 2000). Therefore, while the anatomically-defined region retrosplenial cortex is quite small, the manner in which the label “retrosplenial cortex” is used spans a very large region of the posterior medial cortex with variable cytology (Maguire, 2001; Vogt et al., 2001, 2006) and functional connectivity (Bzdok et al., 2015). This includes the functionally-defined, scene-sensitive “retrosplenial complex” (Epstein, 2008).

Although the mislocalization of the retrosplenial cortex is somewhat egregious, it is not without precedence. Brodmann himself, in fact, intentionally overrepresented the size of the retrosplenial cortex in his original figures (Figure 1A), and he noted this inaccuracy 16 pages after the figures (as it appears in the English translation by Gary). This large representation of the retrosplenial cortex also appeared in the Talairach atlas (Talairach and Tournoux, 1988), with the posterior border of the retrosplenial cortex reaching as far as the junction of the parietal-occipital fissure and the calcarine sulcus. This is in stark contrast to more modern cytological studies, which frequently confirm Brodmann’s original localization (but not depiction) of the retrosplenial cortex as largely contained within the callosal sulcus and without the generous representation on the gyral surface (Figure 1; Morris et al., 2000; Vogt et al., 2001; Fatterpekar et al., 2002).

While it is possible that a slight misrepresentation of an anatomically-defined region provides a more accurate representation of a functionally-defined region involved in spatial orientation and navigation, the “retrosplenial cortex” label has been used in human spatial cognition research far too liberally, including many areas of the posterior medial cortex far beyond the anatomical border of the retrosplenial cortex proper (Nasr et al., 2011; Marchette et al., 2014; Silson et al., 2016). Considering the wide variety of spatial orientation and navigation tasks producing activity in this large area of the human brain, it is likely it could be more accurately described as a collection of relatively distinct subregions, performing slightly different functions within the spatial cognition domain. Consistent with this assumption, we have recently identified a differential engagement of the ventral and dorsal portions of the posterior cingulate cortex while individuals performed a spatial memory task (Burles et al., 2017); we had identified that ventral regions were more involved in updating a mental representation of the environment, and more dorsal regions were involved in recalling the positions of unseen objects from that mental representation. These findings provided initial evidence of a simple encoding-recall specialization along the ventral-dorsal axis of the posterior cingulate and “retrosplenial cortex.” Here, we performed a meta-analysis of relevant fMRI studies to provide further evidence of a ventral-dorsal functional specialization of the posterior cingulate and neighboring cortex supporting the processes of encoding and recalling spatial information.

## Materials and Methods

### Literature Search

To identify relevant neuroimaging studies, we performed a literature search in PubMed identifying fMRI studies with human subjects investigating spatial orientation and mentioning retrosplenial or nearby regions in the posterior medial cortex. We ran the following conjunctive search:

1. retrosplenial OR (posterior cingulate) OR precuneus OR (medial parietal cortex) OR (posterior parietal cortex) OR ((parieto-occipital OR parietooccipital) and (sulcus OR fissure)) OR ((Brodmann Area OR BA) and (23 OR 26 OR 29 OR 30 OR 31))
  AND
2. ((spatial OR topographical OR place OR path OR scene) and (navigation OR memory OR recognition OR learning OR integration OR construction OR imagination OR orientation)) OR path integration OR dead reckoning OR cognitive map OR mental representation OR spatial configuration OR perspective taking
  AND
3. fMRI OR functional magnetic resonance imaging OR functional neuroimaging OR BOLD OR blood oxygen level dependent

This conjunctive search produced 297 articles, which were subsequently filtered to only include the 61 research articles with healthy, adult subjects performing a spatial task while fMRI data were collected, with coordinates reported in the manuscript or supplementary materials. The references in five relevant review articles included in search results were mined, resulting in an additional 23 articles meeting these criteria included from 497 references. Finally, an additional seven articles known to the authors through personal communications with other researchers were included. The total sample of articles passing filtering was comprised of 91 articles. This search strategy was not intended to be exhaustive, but rather generate a sample that is adequately representative of the state of the cognitive neuroscience literature investigating human spatial orientation and navigation.

For each of these 91 articles, we attempted to classify BOLD contrasts as either *spatial encoding* or *spatial recall*. Contrasts classified as *spatial encoding* were principally characterized by relatively more bottom-up or stimulus-driven BOLD activity. These included cases where participants were viewing or imagining visual stimuli, such as landmarks or scenes, or performing active navigation in a novel environment, without an explicit demand to perform a spatial computation or localize unseen landmarks in the environment. For instance, a functional localizer, contrasting BOLD activity while participants viewed scenes over BOLD activity while participants viewed faces or objects (Johnson et al., 2007; Sung et al., 2008) was classified as *spatial encoding*. These contrasts are commonly used to identify scene-sensitive retrosplenial and/or parahippocampal cortex and represent an easily classifiable contrast as the detected BOLD activity relates specifically to encoding scenes and has no demand to recall any spatial or navigational information. This category also included contrasts such as the one performed by Aguirre et al. (1996), subtracting BOLD activity while participants followed an endless, looping corridor from the activity evoked while participants were freely exploring a maze, and presumably encoding the locations of landmarks for a future navigation task.

Conversely, the BOLD contrasts identified as *spatial recall* were generally complementary to the *spatial encoding* category, in a manner similar to a classic encoding—recall dichotomy. For example, in the aforementioned study by Aguirre et al. (1996), free exploration in a maze over a control condition was classified as *spatial encoding*; a complementary contrast of a spatial navigation task (i.e., participants locating a target landmark using the shortest route possible) over a control task, would be classified as *spatial recall*. However, this category also included contrasts weighted more heavily towards spatial representations or judgments in addition to recall *per se*. For instance, Rosenbaum et al. (2004) asked participants to perform proximity judgments between familiar landmarks in downtown Toronto. In this study, landmarks were presented to participants via text, resulting in participants relying strongly on their capacity to recall complex, well-learned, spatial information, and use it to perform a spatial computation, i.e., the proximity judgment.

From the 91 articles passed on to classification, we classified 38 contrasts as *spatial encoding*, and 76 contrasts as *spatial recall* (Supplementary Table S1). We did not classify multiple non-orthogonal contrasts from a single study, and instead selected the contrast most representative of either *spatial encoding* or *spatial recall*, leaving non-orthogonal contrasts unclassified. We then passed all coordinates from classified contrasts to a Multilevel Kernel Density Analysis (MKDA; Wager et al., 2007).

### Multilevel Kernel Density Analysis (MKDA)

We first converted all peak coordinates reported in Talairach space to MNI space (Lancaster et al., 2007), and imported them into NeuroElf^1^ to perform an MKDA. The coordinates were then smoothed using a 12-mm Gaussian kernel and combined to form a single map for each classified contrast, ensuring that contrasts reporting more coordinates (from utilizing more liberal statistical thresholds, for instance) were not overrepresented. These maps were weighted by the square root of the sample size reported in the study. We then compared the z-transformed proportion of voxels differentially and commonly involved in *spatial encoding* and *spatial recall*. To detect differential engagement, we compared the contrast of *spatial encoding vs. spatial recall* against an empirical null distribution generated from label permutation. To detect common engagement, we performed a conjunction from independent activations of *spatial encoding* and *spatial recall* each compared against a spatial scrambling null distribution. In all cases 5000 simulation iterations were performed within an 8385 voxel retrosplenial and posterior cingulate mask, with a 2 mm resolution. The resulting statistical map was thresholded at *p* < 0.001.

### Subregion Functional Connectivity Characterization

To characterize the differences between subregions identified in the MKDA, we contrasted the resting-state functional connectivity profile of regions more likely to be activated by *spatial encoding* contrasts vs. *spatial recall* contrasts and vice-versa. We utilized preprocessed resting state functional connectivity data from 38 unrelated, young adult participants of the Human Connectome Project (Van Essen et al., 2012; Glasser et al., 2013). We performed additional preprocessing on our resting state data using the CONN toolbox (v17.f^2^), modeling head motion with 24 parameters, and regressing out signal from white matter and cerebrospinal fluid (Behzadi et al., 2007), and temporally bandpass filtering between 0.008 Hz and 0.09 Hz. We generated seed regions of equivalent spatial extent from the thresholded results of the MKDA, eroding large clusters of equivalent values by smoothing and re-thresholding. Then, we calculated the difference in functional connectivity displayed by these two subregions by calculating the difference in seed-to-ROI temporal correlation coefficients for 131 ROIs included in CONN’s default atlas, using a statistical threshold of *p*_fdr_ < 0.001. For the connectivity analyses, Fisher-transformed correlation coefficient values were used for comparison, and the reverse transform was applied to return connectivity coefficients to *r* values for ease of interpretation. This research study was approved by the local research ethics board (CHREB-22848).

## Results

### The Retrosplenial Cortex

**Figure 2:**
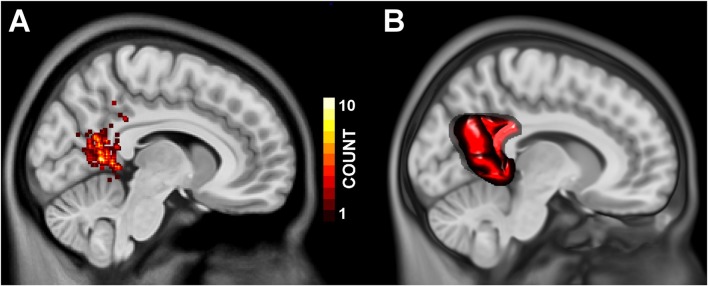
Panel **(A)** depicts the frequency at which a coordinate label included “Retrosplenial Cortex” appeared within 2 mm of any given MNI Y, Z position, projected onto an MNI standard brain at *x* = 8 mm. Panel **(B)** depicts the volume of interest generated to encompass the brain tissue commonly referred to as “retrosplenial cortex” in the spatial cognition literature.

From all 91 articles passing initial filtering, we identified 143 coordinates from 32 articles with a label including “retrosplenial cortex.” Figure 2A depicts a histogram of coordinate locations projected into the sagittal plane. Approximately 10% of reported coordinates lie within the callosal sulcus, i.e., the retrosplenial cortex as defined by Vogt et al. (2001).

### Multilevel Kernel Density Analysis

**Figure 3:**
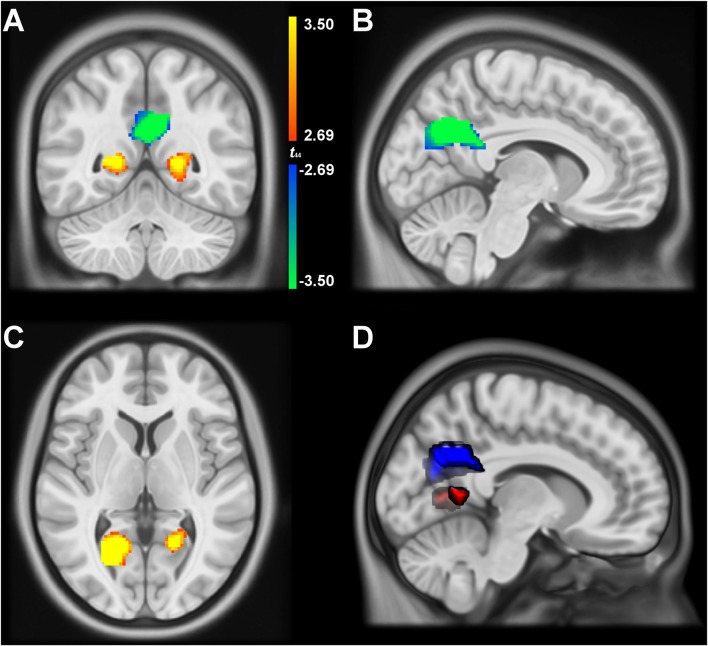
Multilevel kernel density analysis (MKDA) results depicting regions more likely to be activated by *spatial encoding* (red/yellow) and *spatial recall* (blue/green). Panels **(A–C)** are displayed at MNI 8, −53, 5, color range bounds represent uncorrected thresholds of *p* < 0.01 at *t*_(44)_ = 2.69 and *p* < 0.001 at *t*_(44)_ = 3.50 in an 8385-voxel region of interest (Figure 2B). Panel **(D)** displays a volumetric depiction of the significant clusters at *p* < 0.001.

We performed an MKDA to identify regions within the retrosplenial cortex and posterior cingulate (Figure 2B) that are preferentially involved in *spatial encoding* and *spatial recall*. Contrasts classified as *spatial encoding* were generally characterized by stimulus-driven activity in which participants viewed scenes or explored virtual environments, with no explicit demand to localize themselves or unseen landmarks. Contrasts classified as *spatial recall* included those with demands to recall the location of, or route to, landmarks in familiar environments, as well as contrasts that track environmental properties or knowledge (e.g., parametric contrasts with navigational performance or goal proximity). As shown in Figure 3, the MKDA with a threshold of *p* < 0.001 revealed that *spatial encoding* was more likely to activate ventrolateral portions of the posterior cingulate (MNI centroids at −22, −60, 6; 333 voxels, and 20, −56, 6; 70 voxels), whereas *spatial recall* was more likely to activate dorsomedial portions of the posterior cingulate (MNI centroid 4, −60, 28; 847 voxels). These findings closely parallel the results reported in our previous study (Burles et al., 2017). A conjunction analysis did not detect any voxels engaged in both *spatial encoding* and *spatial recall* (peak *t*_(14)_ = 3.719, *p* = 0.002062 at MNI −14, −60, 14).

### Subregion Functional Connectivity Characterization

**Figure 4:**
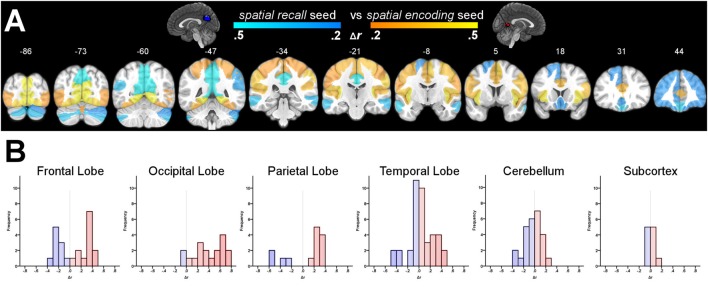
Panel **(A)** depicts the difference in functional connectivity between the ventro-lateral posterior cingulate seeds more associated with *spatial encoding*, and the dorso-medial posterior cingulate seed more associated with *spatial recall*. Highlighted regions display significantly different functional connectivity profiles at *p*_fdr_ < 0.001. *N* = 38. Panel **(B)** displays grouped histograms of the differences in functional connectivity; red highlighting for more positive functional connectivity with the *spatial encoding* seeds, and blue for more positive functional connectivity with the *spatial recall* seed.

From the results of the MKDA, we selected the ventro-lateral clusters totaling 403-voxels more likely to be activated by *spatial encoding*, and a dorso-medial cluster of 408 voxels more likely to be activated by *spatial recall* as seeds for a resting state functional connectivity analysis. Contrasting the functional connectivity profiles of these regions revealed significant differences across the brain, detailed in Supplementary Table S2. Across the 132 brain regions tested, the ventro-lateral and dorso-medial posterior cingulate seeds displayed significantly (*p*_fdr_ < 0.001) different connectivity patterns with 69 regions (i.e., 52% of tested regions). The ventro-lateral *spatial encoding* seed displayed significantly more positive functional connectivity with numerous occipital, lateral parietal, and ventral temporal regions. The dorso-medial *spatial recall* seed, on the other hand, was more positively functionally connected to the posterior cingulate, as well as the frontal pole and dorsomedial prefrontal cortex (see Figure 4).

## Discussion

In the spatial cognition literature, the “retrosplenial cortex” label is used quite liberally for brain regions lying posterior to the splenium of the corpus callosum. Even by Brodmann’s original, overdrawn depiction, a substantial number of MRI peaks labeled “retrosplenial cortex” drawn from the spatial orientation literature lie unequivocally outside of this anatomical region. It is likely that some of the “leaking” of the retrosplenial cortex into the posterior cingulate is not simply due to the rather large representation of the retrosplenial cortex in Brodmann’s work, or the Talairach atlas (Talairach and Tournoux, 1988), but also due to the long history of spatial orientation research in rodents. Rodents lack a clear homologous region to the human posterior cingulate (i.e., BA 23 and 31), and instead boast an expansive retrosplenial cortex (Vogt and Peters, 1981). The human retrosplenial cortex label is applied in a manner that is potentially justifiable as functionally homologous to the rodent retrosplenial cortex, if not anatomically homologous.

While this could be shrugged off as a simple case of difference in nomenclature, we would argue that the lack of specificity in the use of the “retrosplenial cortex” label actively impedes generating a clear and precise understanding of how this region supports the cognitive processes involved in spatial orientation and navigation in humans. In the present study, we provided evidence that the large region that is commonly labeled “retrosplenial cortex” displays a relevant subregion specialization. We classified 114 contrasts from 91 articles as either *spatial encoding* or *spatial recall* and identified that within the “retrosplenial cortex” (more appropriately labeled as the posterior cingulate), ventral portions were more likely to be activated by *spatial encoding*, and dorsal portions more likely to be activated by *spatial recall*.

These findings are supported by a wide variety of previous research that have identified differences in cytology, as well as differences in functional and structural connectivity within this area (Vogt et al., 2006; Hagmann et al., 2008; Zhang and Li, 2012; Bzdok et al., 2015; Silson et al., 2016; Burles et al., 2017), supporting the interpretation that the identified regions are involved in somewhat different cognitive processes. Indeed, we detected markedly different resting state functional connectivity profiles between the ventro-lateral, *spatial encoding*, cluster and the dorso-medial, *spatial recall*, cluster. The *spatial encoding* seeds were centered upon lateral portions of the anterior bank of the common trunk of the parietal-occipital fissure and calcarine sulcus, immediately ventral to where they join. This region displayed more positive functional connectivity coefficients with many ventral-stream, “spatial context” regions, such as the fusiform and lingual gyri (Milner and Goodale, 2008), solidifying its characterization a relatively more involved in bottom-up or lower level perceptual processing and passive updating. In contrast, the *spatial recall* seed was centered 2 cm dorsal to the *spatial encoding* seeds, and displayed relatively greater resting state functional connectivity with regions commonly implicated in spatial manipulation, as well as spatial and episodic memory, such as the posterior cingulate, precuneus and frontal pole (Maddock et al., 2001; Okuda et al., 2003; Ridderinkhof et al., 2004; Wagner et al., 2005; Cavanna and Trimble, 2006; Addis et al., 2009).

While the ventral—dorsal distinction between these subregions was distinct, the *spatial encoding* clusters occupied a relatively more lateral position, deeply tucked within the parietal-occipital fissure. This localization is consistent with previous work by Silson et al. (2016), who localized the scene-sensitive region of the medial parietal cortex as within the parietal-occipital fissure, immediately dorsal to the junction with the calcarine sulcus. This region was characterized by a strong contralateral visual field bias, a property shared with other scene-sensitive cortex (i.e., the occipital and parahippocampal place areas). However, Silson et al. (2016) also described a region immediately anterior and medial to the functionally-localized scene-selective cortex, and noted this region was relatively less scene-sensitive and displayed relatively lower functional connectivity with the posterior parahippocampal place area and occipital place area, but relatively greater functional connectivity with the precuneus, superior frontal, and orbitofrontal cortex. The authors suggest that these regions may constitute partially different scene-processing networks, as proposed by Baldassano et al. (2016). In this paradigm, more lateral scene-sensitive would be relatively more involved in processing visual features, whereas more medial and anterior cortex, approaching or including the retrosplenial cortex proper, appear to be more strongly integrated with the hippocampus and potentially involved in navigation or more general episodic memory processes. Notably, the present meta-analysis did not appear to be sensitive to this region, but this may explain why the *spatial encoding* clusters were sequestered to the lateral portions of the parieto-occipital fissure, as more medial and anterior regions may be involved in processes that are poorly characterized by the *spatial encoding* and *spatial recall* paradigm we adopted.

In conclusion, we believe that the identification of detectable subregions within the posterior cingulate warrants a more precise and nuanced manner in which we discuss and report the results of neuroimaging findings in this region. While the number and location of the particular clusters identified in this meta-analysis likely do not represent *the* relevant subregions of this brain area, we do feel that some simple considerations can be taken into account to reduce the ambiguity of the retrosplenial cortex’s position and role in cognition. First, we would suggest reserving the label “retrosplenial cortex” for peaks which reside within the callosal sulcus, or at least are closer to the callosal sulcus than the parietal-occipital fissure, especially at MNI z positions above +10 mm. Further, for the peaks in the posterior cingulate but in the vicinity of the retrosplenial cortex proper, it may be valuable to begin making the distinction between more ventral and dorsal regions; using the point at which the calcarine sulcus joins with the parietal-occipital fissure as an easily-identifiable landmark for differentiation, or at least reference, as our findings would indicate that regions ventral and significantly dorsal to this point may not be functionally homogenous.

## Author Contributions

FB and GI conceived and designed the study. FB, AU, LHM and KP collected and analyzed the data. All authors contributed to and have approved the final manuscript.

## Conflict of Interest Statement

The authors declare that the research was conducted in the absence of any commercial or financial relationships that could be construed as a potential conflict of interest.

## Footnotes

^1^neuroelf.net/

^2^nitrc.org/projects/conn
